# Soft-Surface DNA Nanotechnology: DNA Constructs Anchored and Aligned to Lipid Membrane**

**DOI:** 10.1002/anie.201103338

**Published:** 2011-07-14

**Authors:** Karl Börjesson, Erik P Lundberg, Jakob G Woller, Bengt Nordén, Bo Albinsson

**Affiliations:** Department of Chemical and Biological Engineering, Chalmers University of Technology41296 Göteborg (Sweden)

**Keywords:** DNA structures, liposomes, nanotechnology, porphyrinoids, self-assembly

Two-dimensional (2D) DNA assemblies are usually, for imaging purposes, immobilized on hard surfaces resulting in irreversible systems devoid of any dynamics. Herein, the attachment of a 2D DNA nanoconstruct to a soft lipid membrane surface is reported. The membrane anchor is a porphyrin nucleoside and at least three attachment points are needed to align the DNA nanoconstruct onto the membrane surface. This attachment methodology results in freely diffusing DNA constructs that reversibly can be assembled on the surface enabling the possibility of a self repairing system.

DNA is attractive as a building block in bottom-up approaches of nanotechnology. The small size of the double helix, 2 nm width, together with its stiffness offers a material with high spatial resolution potential. The strongly selective hydrogen-bonding pattern of the Watson & Crick base pairs enables assembly of multidimensional structures from 1D DNA molecules in a rational manner. The thermal reversibility of formation of double-stranded DNA offers the possibility to assemble/disassemble a complex an infinite number of times using easily accessible temperatures (20–80 °C), and furthermore, the structure can be made so that only parts of the complex are disassembled with a small increase in temperature. These are the prime attributes together with the low cost and ease of modification that has made DNA popular in nanotechnological contexts.

2D structures of DNA have been built in various shapes and bound to hard surfaces (typically mica).[1] These surface-bound constructs offer the possibility to be used as templates for heteromaterials on the nanoscale and as functionalized grids. However, when bound to hard surfaces with electrostatic forces, the constructs lose the ability to freely diffuse and thus rearrange on the surface. Constructs in solution, on the other hand, still retain all of the natural features of DNA such as addressability for site-specific functionalizations.[2] However, for many applications, for example, nanoelectronics, the communication with a surface is of prime importance.

We have tried to solve this contradiction of having the DNA on a surface for application purposes together with having it in solution to retain all of its supramolecular abilities by using porphyrin anchors. The anchoring force is the hydrophobic affinity of the porphyrin to the interior of the membrane. This soft attachment enables lateral diffusion of the anchored DNA on the surface. Moreover, it facilitates a reversible assembly of the system on the membrane, thus giving rise to the possibility of self-repair by a simple heating/cooling cycle. The porphyrin-anchored DNA network looks much like a power grid, where the porphyrins, like poles, hold the grid in place. This concept allows the DNA to be fully accessible from the solution while being close enough to the surface to be able to communicate with it, thus bridging the 3D space of the solution with the 2D space of the membrane surface. The porphyrin moiety is not merely an anchor, but also a multifunctional unit with both the ability to communicate with the surface and probe the binding strength to the membrane, features we have investigated in previous work.[3]

**Figure 1:**
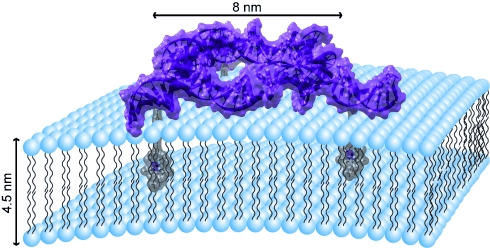
Schematic picture of the DNA network anchored to a lipid membrane using three porphyrins (HexP3). The large radius of curvature of the liposome results in a virtually flat surface onto which the DNA construct binds.

The 2D DNA structures used in this study are based on a hexagonal core with three protruding arms, 120 degrees from each other (Figure 1). The hexagonal core, with each side being 10 bases long, is made up of six 22-mer oligonucleotides. The sequence of the 22 bases is designed to have two stretches of 10 bases separated by two thymines. Each stretch of 10 bases has one unique partner in the system, thus forming a side in the hexagonal core. The two thymines are unpaired, thereby functioning as hinges to give the system bending flexibility. Physical properties of this hexagonal-like DNA nanostructure have been well studied in our laboratory.[4] The three protruding arms are stretches of 39 bases, complementary to the porphyrin-modified oligonucleotide (the oligonucleotide synthesis is described in detail elsewhere[3b]). The arms are situated in the 5′ end of three 22-mer oligonucleotides in the hexagonal core, with two unpaired thymines as flexibility spacers (information about sequences can be found in the Supporting Information). Upon hybridization, the porphyrin anchors are located on the arms close to the hexagonal core, and structures with porphyrins on one (HexP1), two (HexP2), or on all three (HexP3) arms have been constructed to investigate the effect and binding geometry of the membrane-tethered DNA nanostructure.

**Figure 2:**
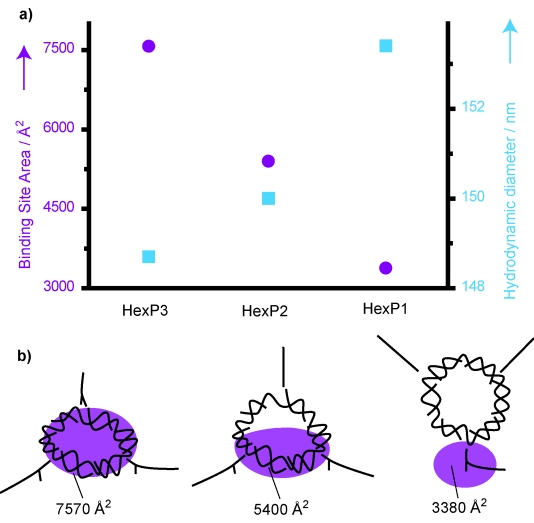
a) The binding site area of the hexagonal-like 2D DNA (purple circles) and the diameter of the liposome–DNA construct (blue squares) are shown as a function of the number of anchoring points. The diameter of liposomes in the absence of DNA was 130 nm. b) An interpretation of the data is shown: whereas HexP3 is lying flat on the surface, HexP1 is more perpendicular to the surface, and HexP2 is somewhere in between.

The primary attachment force that the DNA structures experience with the lipid membranes is the one of the porphyrin anchors. Therefore, it is plausible to expect that the singly anchored structure (HexP1) will be in a different conformation relative to the membrane surface as compared to the triply anchored structure (HexP3). Indeed this is so, and Figure 2 a presents the size of the binding site as measured by photometric titration (examination of the porphyrin emission with increasing concentration of liposomes; see the Supporting Information). The estimated surface coverage of HexP3 is approximately the size of the hexagon ring itself, whereas HexP1 requires only less than half of that space on the surface. The natural explanation for this is that HexP3 is located in a close to tangential orientation on the surface whereas HexP1 is in a more loose conformation, probably projecting more or less perpendicular to the surface. HexP2, having a binding site that has a size between that of HexP1 and HexP3, probably also has a looser positioning than HexP3, but not as loose as HexP1.

To confirm that HexP1 is located in a more perpendicular position than HexP3, dynamic light scattering (DLS) was performed on fully DNA-saturated liposomes. The porphyrin DNA constructs are expected to affect the liposome size in two ways. Firstly, the porphyrin moieties are located inside the membrane, and thus, add a substantial amount of mass to the membrane forcing it to expand. This effect should be largest for HexP3 since it has the highest concentration of porphyrins in the membrane. Secondly, the DNA and the conformation it has on the surface should affect the size of the liposomes. This effect ought to display the opposite trend and yield a bigger sphere for HexP1 where more of the DNA structure may stretch out from the membrane surface. The DLS data presented in Figure 2 a shows a larger hydrodynamic diameter for HexP1 compared to HexP2 and even more so compared to HexP3. This result clearly shows that the protruding DNA on the outside of the membrane is the predominant cause of the size increase and supports the conclusion that the singly anchored DNA nanostructure is in a more perpendicular position to the membrane than the other two constructs (Figure 2 b).

**Figure 3:**
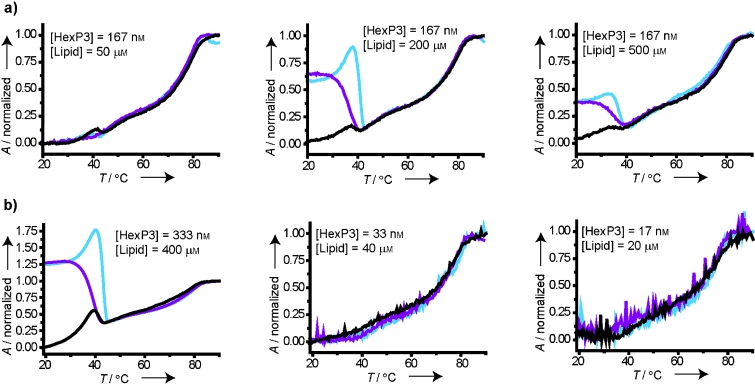
Melting curves (absorption at 260 nm (DNA) as a function of temperature) of HexP3 prehybridized in buffer whereafter liposomes were added (first temperature rise: black, temperature decrease: purple, and second temperature rise: blue). a) Melting curves with constant DNA concentration. b) Melting curves with constant DNA/liposome ratio but at different total concentrations.

One of the advantages of having a DNA construct attached to a surface through a spacer, instead of electrostatic forces working on the DNA itself, is that the DNA construct can be reversibly assembled on the surface. Figure 3 shows melting curves (absorbance at 260 nm) for HexP3 at various DNA and lipid concentrations. These constructs have two distinct melting regions, the hexagonal core that melts at about 40 °C and the arms on which the porphyrins are located that melts at about 80 °C (see the Supporting Information for melting curves for HexP1, HexP2, and for unmodified construct). The constructs in Figure 3 are prehybridized in the absence of liposomes, whereafter liposomes were added and the melting experiments performed. Note that when the DNA melts on the lipid bilayer, the porphyrin-modified oligonucleotide is still attached to the membrane whereas the unmodified DNA is not. The most striking effect in the melting curves of HexP3 (but not for HexP2 or HexP1) is the lack of reversibility at some concentrations. When the constructs are rehybridized on the lipid membrane an increase in the optical density (OD) is seen. Surprisingly, when these samples are heated up again a rise in OD is seen first and then a lowering in OD (relative to that of the first melting) is observed. This transition occurs in the temperature region where the hexagon core melts. The reason for this behavior is that the HexP3 construct has three anchor points and has the possibility to link separate liposomes together with the hexagonal structure acting as a bridge. Forming linked structures will increase the OD because bigger aggregates scatter more light. This effect looks very similar, to the melting curves of a DNA controlled assembly of liposomes in a recent study by Vogel and co-workers.[5] DLS also supports the hypothesis that larger aggregates are formed reversibly (see the Supporting Information). Interestingly, in our case this unwanted interaction can be avoided in two ways. Firstly, by changing the DNA/lipid ratio. In Figure 3 a the DNA concentration is kept constant whereas the lipid concentration is increased, going from left to right. At 200 and 500 μm concentrations of lipid the irreversible hybridization, seen as an increase in OD at lower temperatures, is clearly observed. However, at 50 μm concentration of lipids (left) there is no light scattering effect and the melting process is totally reversible. At this lower lipid concentration the DNA is more closely packed on the surface, leading to a much higher local concentration of DNA on the lipid membrane. This means that even if diffusion is slow on the surface, the correct DNA parts making up the hexagon have higher probability to meet each other on the same liposome compared to hybridizing with complementary parts on another liposome. In Figure 3 b the ratio of DNA to liposomes is constant, but the entire system is more and more dilute, going from left to right. This means that the concentration of bound DNA on the liposome surface will be the same for all samples.[6] However, the dilution of the system will affect the average liposome-to-liposome distance and thus, the frequency of liposome-to-liposome encounters. By diluting the system the number of liposome-to-liposome contacts will decrease. As predicted, by lowering the total concentration the light scattering effect disappears. Thus, it is the competition of diffusion on the membrane surface versus the diffusion of the liposomes that governs if the DNA constructs will link liposomes together. DLS was performed to verify that the increase in OD is a light scattering effect based on increased size of the particles in solution (see the Supporting Information).

**Figure 4:**
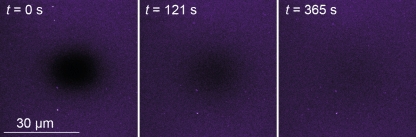
Three snapshots from a FRAP series of Cy5-tagged HexP3 attached to a supported lipid bilayer directly after bleaching (*t*a = 0 s), at an intermediate time (*t*a = 121 s), and after full recovery (*t*a = 365 s).

To explore hexagon diffusion on the membrane surface, fluorescence recovery after photobleaching (FRAP) was performed. Hexagons having 1–3 porphyrin anchors were attached to supported bilayers and a Cy5 tag was used to probe the emission from the surface-bound constructs. In Figure 4, three pictures from a FRAP series on HexP3 are seen (see the Supporting Information for FRAP snapshots and Hankel-transformed fluorescence recovery data on all three systems). They clearly show that the DNA construct diffuses into the bleached spot from the sides, and that the bleached spot is fully restored with time. This does not only give additional proof that the hexagons are anchored to the surface but also shows that the structures are freely diffusing on the surface. The FRAP data was analyzed using the Hankel transform method, which transforms the fluorescence recovery into the frequency domain.[7] The diffusion constants were determined to be 0.6, 0.9, and 2 μm^2^ s^−1^ for HexP3, HexP2, and HexP1, respectively. Thus, the rate of diffusion depends on the number of anchor points to the surface. This is expected since each anchor point inflicts friction to the surface slowing down the diffusion.

In conclusion, we have tethered a hexagonal DNA nanostructure to a lipid membrane using multifunctional porphyrin anchors. Unlike attachments to solid supports, this system is dynamic and allows the DNA to retain all of its attractive solution properties. We have shown that multiple anchor points are needed to hold the construct in place on the surface and furthermore that the reversibility of the assembly on the surface is very much related to the diffusion of the anchored DNA strands in comparison to the diffusion of the liposomes. Finally, we have taken the first step to a soft attachment methodology for DNA nanoconstructs, which enables lateral diffusion on the surface, reversibility of the assembly, and the possibility of a heat-induced self-repair mechanism of the DNA nanoconstructs.
