# Supplementary material for: Soft-Surface DNA Nanotechnology: DNA Constructs Anchored and Aligned to Lipid Membrane
Source: Angew Chem Int Ed Engl. 2011 Jul 14;50(36):8312–5. doi: 10.1002/anie.201103338 (PMC3193381; doi:10.1002/anie.201103338)
Supplement: Supplementary file 1 [file anie0050-8312-SD1.pdf]

Angewandte Chemie
Eine Zeitschrift der Gesellschaft Deutscher Chemiker

Supporting Information
© Wiley-VCH 2011
69451 Weinheim, Germany

Soft-Surface DNA Nanotechnology: DNA Constructs Anchored and Aligned to Lipid Membrane**

*Karl Börjesson, Erik P. Lundberg, Jakob G. Woller, Bengt Nordén, and Bo Albinsson**

anie_201103338_sm_miscellaneous_information.pdf

Supporting Information

**S2**	**DNA sequences**
**S3**	**Details for liposome preparation, photophysical and FRAP measurements**
**S5**	**Additional melting curves**
**S6**	**Binding isotherm and fitted data for liposome titration**
**S7**	**DLS data on HexP3 hybridized on liposome surface at different total concentrations**
**S8**	**Hankel transformed recovery data and additional FRAP snap shots**
**S8**	**References**

DNA sequences

Oligonucleotides were purchased from ATDbio with the exception of the porphyrin modified oligonucleotide that was synthesized and characterized as previously described.[1]

Table S1. Sequences used in the study (color coded to Figure S1). The first three sequences were Cy5 tagged in the FRAP experiments.

CCATACATACTTCCACAGCATC
GGCTCTACAGTTGAGGAGGATG
GATTAGCGTCTTCGATGGTATC
CGTCTGAGTGTGTCTAGCTGATTGGTTGGGATTGCGGCCTTGACGCTAATCTTGATGCTGTGG
CGTCTGAGTGTGTCTAGCTGATTGGTTGGGATTGCGGCCTTGTATGTATGGTTCATCCTCCTC
CGTCTGAGTGTGTCTAGCTGATTGGTTGGGATTGCGGCCTTCTGTAGAGCCTTGATACCATCG
CGGACTATGCTCGTTCAGTTCTGTCTCTTGTGTTCGATGTTGTATGTATGGTTCATCCTCCTC
CGGACTATGCTCGTTCAGTTCTGTCTCTTGTGTTCGATGTTCTGTAGAGCCTTGATACCATCG
CATCGAACACAAGAGACAGAACTGAACGAGCATAGTCCG
GGCCGCAAT^{porphyrin}CCCAACCAATCAGCTAGACACACTCAGACG
GGCCGCAATLPCCCAACCAATCAGCTAGACACACTCAGACG

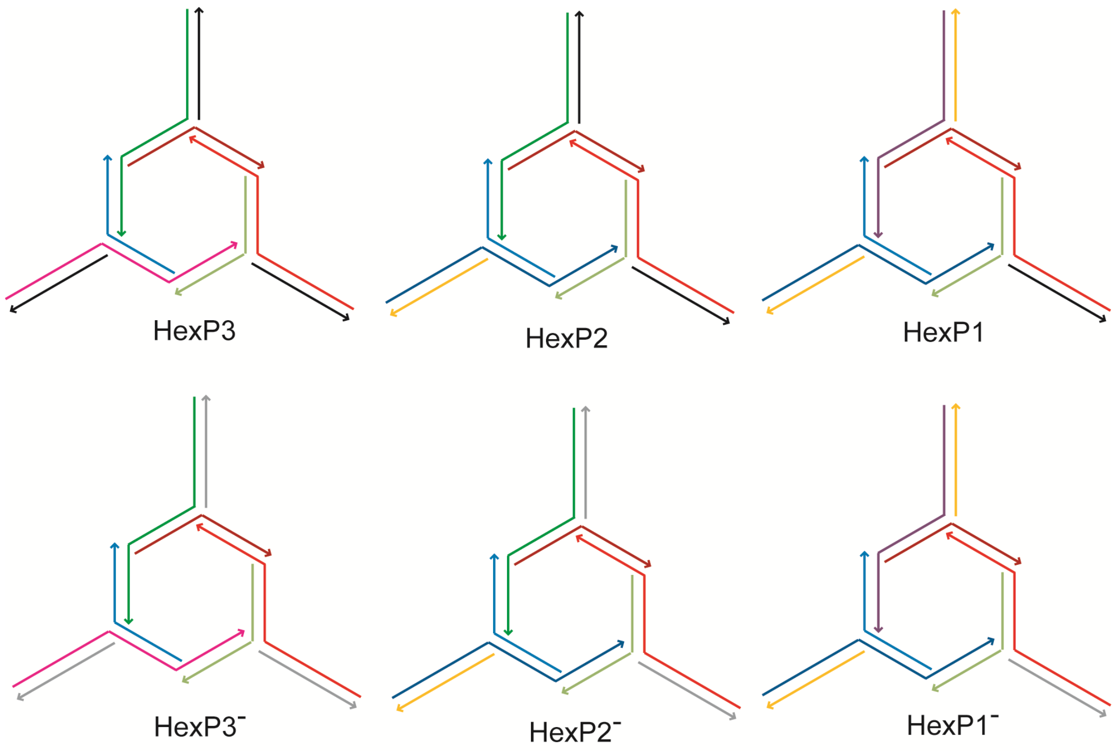

Figure S1. DNA structures used in the study, color coded as in Table S1. The minus sign indicates structures without any porphyrins (negative control).

Liposome preparation

1,2-Dioleoyl-*sn*-glycero-3-phosphocholine (DOPC) was purchased from Avanti Polar Lipids. Large unilamellar vesicles (LUVs) were prepared by standard procedure as briefly described here. A thin lipid film was created by evaporating a chloroform solution of the lipids, which was subsequently dissolved in aqueous buffer and subjected to freeze-thaw cycling (5 times). The solution was then extruded 21 times through 100 nm polycarbonate filters (Whatman). Dynamic light-scattering (DLS) was used to confirm the size of the liposomes and liposome-DNA constructs. DLS was performed on a Malvern Zetasizer Nano ZS.

Photophysical measurements

All measurements were made in a TRIS-HCl buffer at pH 8 in total sodium ion and TRIS concentrations of 500 mM and 25 mM, respectively. Absorption spectra were measured on a Varian Cary 4000 spectrophotometer. Steady state fluorescence spectra were measured on a Spex Fluorolog 3 spectrofluorimeter (JY Horiba) by exciting the samples at 543 nm.

Liposome titration: The binding constants of the porphyrin-DNA complexes to liposomes were determined by spectrophotometric titration. The titrations were performed by adding aliquots of a liposome stock solution to the porphyrin-DNA adduct solution. The concentrations of hexagons were 333 nM at start. Corrections for the volume change were made in the subsequent analysis. The analysis was based on the standard equilibrium equation:

$$K = \frac{[HexP_{Bound}]}{[HexP_{Water}][free\ binding\ sites]} = \frac{[HexP_{Bound}]}{[HexP_{Water}]([L_0]\bullet n - [HexP_{Bound}])} \qquad \text{Eq. S1}$$

Where *[HexP]* is the concentration of DNA-porphyrin complex in water or bound to liposomes, $[L_0]$ is the concentration of lipids in solution and *n* is the size of the binding site in binding sites/lipids. The equilibrium equation can be rearranged to express the total amount of liposome-bound porphyrin:

$$[HexP_{Bound}] = \frac{\left(\frac{1}{K} + [HexP_{Tot}] + n[L_0]\right)}{2} - \sqrt{\frac{\left(\frac{1}{K} + [HexP_{Tot}] + n[L_0]\right)^2}{4} - [L_0][HexP_{Tot}]n} \qquad \text{Eq. S2}$$

where *[HexP$_{Tot}$]* is the total porphyrin concentration. In the analysis, the binding was assumed to go to completion at high lipid concentration, and at intermediate lipid concentrations, the spectra were treated as a linear combination of the unbound and bound states.

Fluorescence recovery after photobleaching

Glass slides (25 mm diameter, #0-1) were cleaned in 10 mM SDS solution for >24 h, rinsed thoroughly with milli-Q water, dried under N_2 flow, and treated with UV-ozone for 45 min. Cleaned glass slides were mounted in an in-house made flow cell. Buffer and subsequently 0.1 mg/ml DOPC vesicles in buffer were flowed over the surface and left to stand for 0.5-1 h for formation of supported lipid bilayer to take place. The glass slides were rinsed with buffer to remove excess of vesicles. Solution (concentration 167 nM) of Cy5 labeled hexagons was flowed over the surface and left to equilibrate for 5 min (repeated 5 times), where after unbound hexagons were removed with a buffer rinse. Finally, the flow cell was mounted in the microscope. FRAP data were obtained using a Leica TCS SP confocal laser scanning microscope with a 63X magnification Leica oil immersion objective (NA=1.23), and triple dichroic filter (TD 488/568/647). The samples were excited and bleached using a 633 nm laser line. FRAP data from different regions of the sample were collected in order to improve statistics. FRAP data were analyzed using the Hankel transform method, using a freely available MatLab script.[2]

Additional melting curves

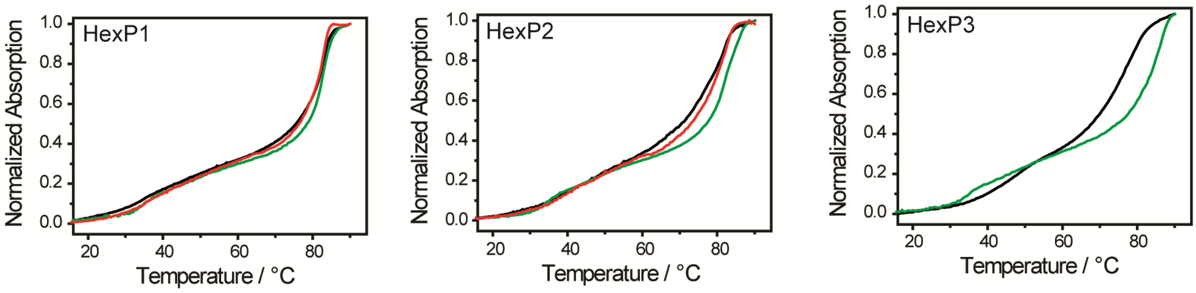

Figure S2. Absorption melting curves for hexagonal like 2D DNA in buffered solution (black), in presence of liposomes (red) and negative control without the porphyrin anchors (green). Melting curves of HexP3 in presence of liposomes, measured at various DNA and liposome concentrations, are shown in Figure 3.

Binding isotherm and fitted data for liposome titration

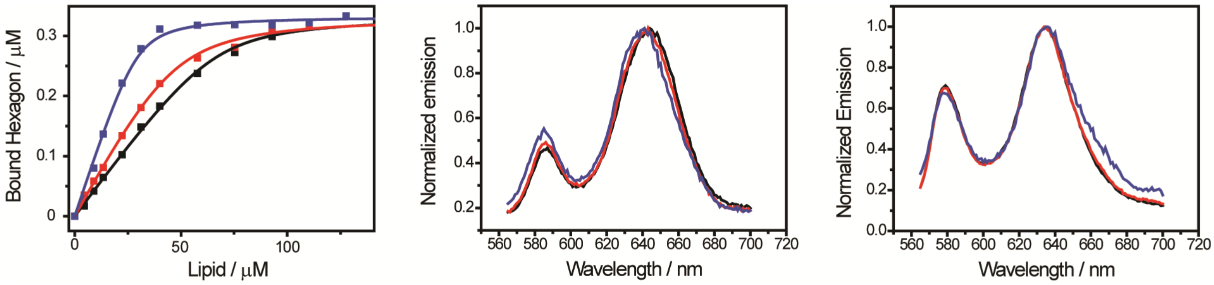

Figure S3. Left: Binding isotherms for complexes with one (blue), two (red) and three (black) porphyrin anchors. Concentration bound complexes (squares measured data and line best fit of data to Equation S2) as a function of lipid concentration. Middle: Normalized emission spectra of the starting point in the titration. Right: Normalized emission spectra of the endpoint in the titration.

Table S2. Binding constant (M^{-1} lipid) and binding site area of porphyrin-DNA complexes. For analysis procedure see photophysical measurement section or reference 1.

	Binding constant (M^{-1})[a]	Binding site size (binding site/lipid)	Binding site area ($Å^2$)[b]
HexP3	6•10^7	0.0050	7570
HexP2	3•10^7	0.0070	5400
HexP1	6•10^7	0.0112	3380

[a]The DNA concentration used resulted in very shallow residuals for the binding constants.
[b]Based on 52.4 % of lipid mass in the outer leaflet and 72.2 $Å^2$/lipid.[3]

Dynamic Light Scattering

Dynamic light scattering (DLS) was performed to confirm that the increased optical density seen at low temperatures in some of the absorption melting curves indeed is a light scattering effect due to increased size of the liposome DNA complexes. In Table S3 the diameter (z-average) of two samples (corresponding to Figure 3b right and left) with the same DNA to lipid ratio but hybridized at different total concentrations can be seen. Prior to DLS measurement the concentration was adjusted so both samples had equal concentration of DNA and lipid, and thus, the only difference between them was the concentration when hybridized. There is a 10 nm difference in diameter between the two samples, suggesting that the increased optical density seen at low temperatures is an effect of larger aggregates. Furthermore, when increasing the temperature above the melting temperature, the difference in diameter due to hybridization procedure disappears, and when hybridized again, the two samples show the same diameter corresponding to the non-aggregated state. Thus, the aggregation can be removed by dilution and rehybridization.

Table S3. Dynamic light scattering data on HexP3 hybridized on the liposome surface at different concentrations. Average diameter (z) and polydispersity index (PDI) are given through a melting-rehybridization cycle.

Concentration at hybridization[a] (nM)	z (PDI) at 15 °C (nm)	z (PDI) at 45 °C (nm)	z (PDI) at 15 °C after cooling (nm)
[HexP3]=333[b]	142.2 (0.12)	138.5 (0.087)	133.1 (0.105)
[HexP3]=17	132.9 (0.075)	139.1 (0.084)	131.5 (0.117)

[a]The lipid DNA ratio was 1200 in all cases.

[b]The sample was hybridized at 333 nM but diluted 20 times prior to DLS measurement, resulting in a HexP3 concentration of 17 nM.

Hankel transformed recovery data and additional FRAP snap shots

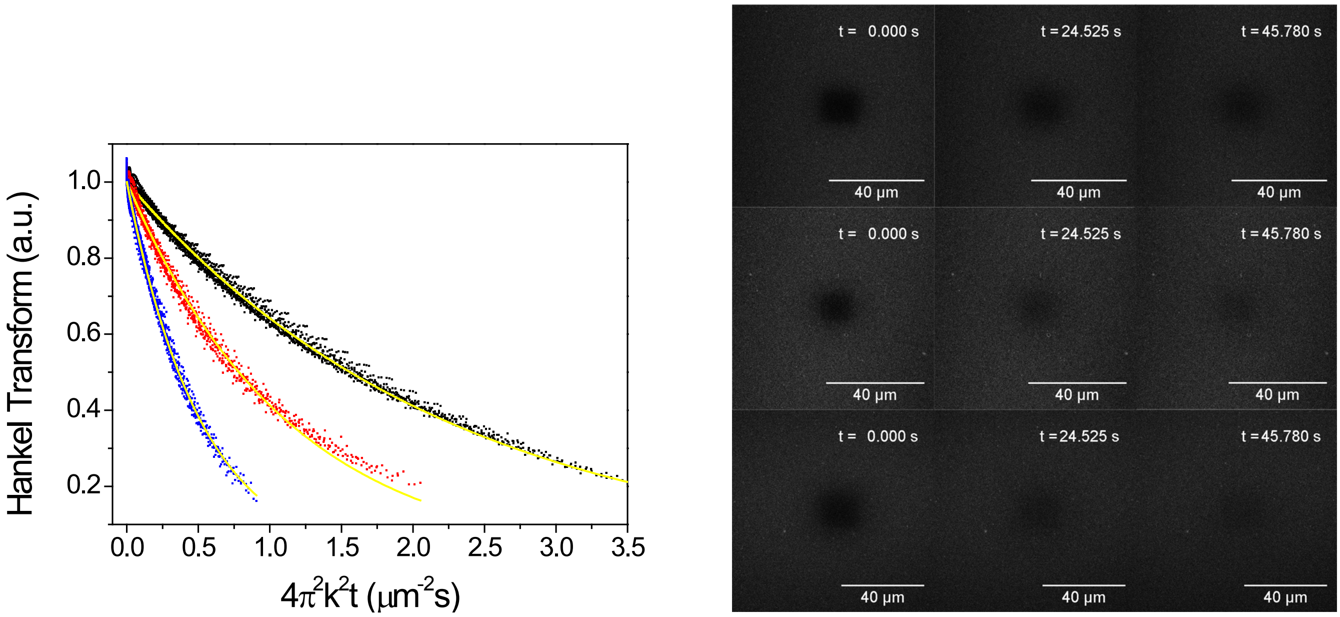

Figure S4. Left: Hankel transformed recovery data for HexP3 (black), HexP2 (red) and HexP1 (blue). Best fit is shown as a solid yellow line. Right: Three snap shots from FRAP series of Cy5 tagged HexP3 (top), HexP2 (middle) and HexP1 (bottom) attached to a supported lipid bilayer. The time scales indicate time after bleach.

References

[1] K. Börjesson, J. Wiberg, A. H. El-Sagheer, T. Ljungdahl, J. Mårtensson, T. Brown, B. Nordén, B. Albinsson, *ACS Nano* **2010**, *4*, 5037.

[2] a) http://www.mathworks.com/matlabcentral/fileexchange/29388-frapanalysis; b) P. Jönsson, M. P. Jonsson, J. O. Tegenfeldt, F. Höök, *Biophys. J.* **2008**, *95*, 5334.

[3] S. Tristram-Nagle, H. I. Petrache, J. F. Nagle, *Biophys. J.* **1998**, *75*, 917.
